# Expanded geographic distribution and host preference of *Anopheles gibbinsi* (*Anopheles* species 6) in northern Zambia

**DOI:** 10.1186/s12936-022-04231-5

**Published:** 2022-07-03

**Authors:** Mary E. Gebhardt, Rachel S. Krizek, Maureen Coetzee, Lizette L. Koekemoer, Yael Dahan-Moss, David Mbewe, James Sichivula Lupiya, Mbanga Muleba, Jennifer C. Stevenson, William J. Moss, Douglas E. Norris, For the Southern Africa International Centers of Excellence for Malaria Research

**Affiliations:** 1 The W. Harry Feinstone Department of Molecular Microbiology and Immunology, Johns Hopkins Malaria Research Institute, Johns Hopkins Bloomberg School of Public Health, Baltimore, MD USA; 2 Wits Research Institute for Malaria, Faculty of Health Sciences, University of the Witwatersrand, and the Centre for Emerging Zoonotic & Parasitic Diseases, Vector Control Reference Laboratory, National Institute for Communicable Diseases of the National Health Laboratory Service, Johannesburg, South Africa; 3 Tropical Diseases Research Centre, Ndola, Zambia; 4 Macha Research Trust, Choma, Zambia; 5 Department of Epidemiology, Johns Hopkins Bloomberg School of Public Health, Baltimore, MD USA

**Keywords:** Malaria, Vectors, Anopheles gibbinsi, Anopheles species 6, Zambia, ICEMR

## Abstract

**Background:**

Nchelenge District in northern Zambia suffers from holoendemic malaria transmission despite a decade of yearly indoor residual spraying (IRS) and insecticide-treated net (ITN) distributions. One hypothesis for this lack of impact is that some vectors in the area may forage in the early evening or outdoors. *Anopheles gibbinsi* specimens were identified in early evening mosquito collections performed in this study area, and further insight was gleaned into this taxon, including characterizing its genetic identity, feeding preferences, and potential role as a malaria vector.

**Methods:**

Mosquitoes were collected in July and August 2019 by CDC light traps in Nchelenge District in indoor sitting rooms, outdoor gathering spaces, and animal pens from 16:00–22:00. Host detection by PCR, COI and ITS2 PCR, and circumsporozoite (CSP) ELISA were performed on all samples morphologically identified as *An. gibbinsi,* and a subset of specimens were selected for COI and ITS2 sequencing. To determine risk factors for increased abundance of *An. gibbinsi,* a negative binomial generalized linear mixed-effects model was performed with household-level variables of interest.

**Results:**

Comparison of COI and ITS2 *An. gibbinsi* reference sequences to the NCBI database revealed > 99% identity to “*Anopheles* sp. 6” from Kenya. More than 97% of specimens were morphologically and molecularly consistent with *An. gibbinsi*. Specimens were primarily collected in animal pen traps (59.2%), followed by traps outdoors near where humans gather (24.3%), and traps set indoors (16.5%). Host DNA detection revealed a high propensity for goats, but 5% of specimens with detected host DNA had fed on humans. No specimens were positive for *Plasmodium falciparum* sporozoites. Animal pens and inland households > 3 km from Lake Mweru were both associated with increased *An. gibbinsi* abundance.

**Conclusions:**

This is the first report of *An. gibbinsi* in Nchelenge District, Zambia. This study provided a species identity for unknown “*An*. *sp. 6*” in the NCBI database*,* which has been implicated in malaria transmission in Kenya. Composite data suggest that this species is largely zoophilic and exophilic, but comes into contact with humans and the malaria parasites they carry. This species should continue to be monitored in Zambia and neighbouring countries as a potential malaria vector.

**Supplementary Information:**

The online version contains supplementary material available at 10.1186/s12936-022-04231-5.

## Background

After 15 years of global reductions in malaria cases, progress has slowed since 2015, with an estimated 229 million malaria cases in 2019 [1]. Progress made has been heterogeneous across Africa, with some countries still experiencing a high burden of malaria, and others approaching elimination. In regions where major vectors have been successfully controlled, species that were once secondary vectors or had remained unrecognized have become an increasing concern, and may be responsible for more transmission than previously documented [2–8]. Many of these anopheline species tend to be exophilic or display behavioural plasticity in their foraging and resting behaviours, and may not choose humans as their preferential host [9, 10]. It is unclear if these species always contributed to transmission, or if they fill an open niche when primary vector populations are reduced [4, 5, 8, 10–12]. To fully understand the importance of these implicated vectors, their roles in transmission must be characterized not only when a region is close to elimination but when other well-studied anophelines are primarily driving transmission.

Nchelenge District is located in Luapula Province, northern Zambia and lies along the eastern shore of Lake Mweru. Many streams and tributaries in this district empty into the lake, creating a marshy ecology for malaria vector breeding sites that drives high year-round malaria transmission [13–16]. Well-recognized vectors in the area include *Anopheles funestus *sensu stricto (*s.s*.)*,* with peak abundance during the dry season, and *Anopheles gambiae s.s.* with persistent low-level abundance. Both species reproduce year-round in this region [14]. High transmission in Nchelenge District has continued even after annual indoor residual house spraying (IRS) and multiple mass insecticide treated net (ITN) campaigns across the district, leading to the hypothesis that vectors may be biting outside or before people go under their mosquito nets at night [15–18].

To better characterize additional potential vectors, mosquito collections were performed to identify early-foraging anopheline species in Nchelenge District, Zambia, during the cool dry season in 2019. A large proportion of specimens collected as part of this study were *Anopheles gibbinsi*, a species which has not previously been reported in Zambia. Literature on this species is sparse, though it has been documented in the highlands of eastern Africa, from Ethiopia down to the Democratic Republic of the Congo (DRC) [2, 19–23]. Importantly, 7.7% of morphologically-identified *An. gibbinsi* captured in an early-foraging study in Kenya were determined to be positive for *Plasmodium falciparum* sporozoites, suggesting that this species is a vector of malaria [2]. In this study, further insight was gleaned into this understudied species, including characterizing its genetic identity, feeding preferences, and potential role as a malaria vector.

## Methods

### Study area and household selection

**Fig. 1:**
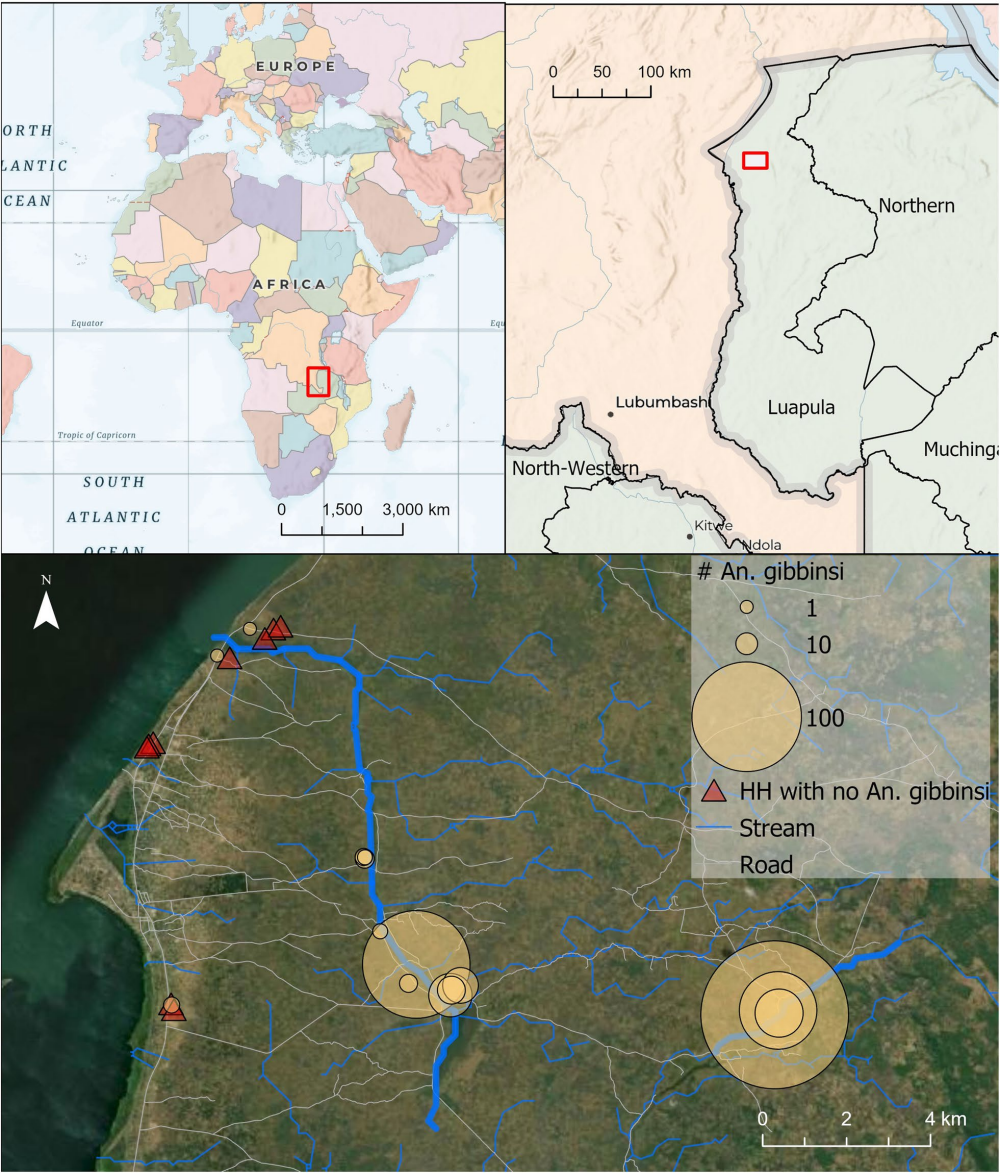
Top panels indicate the geographic location of the study site. Yellow circles in the bottom panel represent the total counts of *An. gibbinsi* captured at each household throughout the study period. Households with no *An. gibbinsi* are represented by red triangles

Data were collected in July and August 2019 in Nchelenge District, Zambia (Fig. 1). This region experiences three seasons: a rainy season from December to April, a cold dry season from May to August, and a hot dry season from September to November [14]. Nchelenge District lies along Lake Mweru and has several streams and tributaries that persist during the dry season, creating an expanse of marshy mosquito breeding sites.

Twenty-four households were selected for this study: twelve located < 3 km from the lake’s shore and twelve located > 3 km from the lake (lakeside and inland, respectively). Sampled households were required to have at least one animal pen outside of the main sleeping structure, and inland households were required to be located within 0.6 km of a major stream.

### Environmental covariates

During the study visits, household coordinates were uploaded into ArcGIS Pro (ESRI, Redlands, CA, USA). Streams were previously mapped and categorized [17], and distance to the nearest major stream was calculated using the “near” tool in ArcGIS Pro (ESRI, Redlands, CA, USA).

### Entomological sampling

Three lakeside and three inland households were sampled each day, and a Latin-square method was used to rotate the trap placement in each sampled household [24]. Mosquitoes were collected using miniature CDC Light Traps (John W. Hock Co., Gainesville) from 16:00 to 22:00. The head of household was provided a watch at the time of set up and was asked to turn the trap on at 16:00 and tie the collection bag at 22:00. Traps were hung between 1.5: 1.8 m above the ground, either in the indoor sitting room of a household, an outdoor area where people gather in the evening, or outdoors next to an animal pen. Other than the incandescent light on the trap and any people or animals that spent time near the trap, no other lures were used. Surveys regarding household features and human behaviour were conducted at the time of enrollment and additionally for each trap collection. The head of household answered all the enrollment questions, and if they were not present for the follow-up questionnaire, the spouse or eldest child responded.

### Mosquito processing

Captured mosquitoes were killed by freezing, morphologically identified using Gillies and Coetzee [25], and stored individually in a microfuge tube on silica gel to desiccate in the field. Desiccated samples were returned to the Johns Hopkins Bloomberg School of Public Health (BSPH) in Baltimore, Maryland, USA, where each specimen was split into head/thorax and abdomen. Prior to splitting, 17 samples that were morphologically identified as *An. gibbinsi* were sent to the University of the Witwatersrand to confirm morphological identity.

DNA from female anopheline mosquito abdomens were crushed in lysis buffer using a Qiagen TissueLyser II (Qiagen, Hilden, Germany) and extracted using an automated DNA extraction method with a QIACube HT (Qiagen, Hilden, Germany) at Purdue University [26]. After DNA extraction, each specimen underwent a PCR assay to amplify a fragment of the internal transcribed spacer region 2 (ITS2) of the nuclear genome as described previously [27–29]. A random representative subset of 5.4% of *An. gibbinsi* specimens were selected for Sanger sequencing of the ITS2 target and the Barcode of Life fragment of the cytochrome oxidase I (COI) gene as previously described [27–29]. Sequencing for specimens returned to BSPH was conducted at the Johns Hopkins Medical Institutions (JHMI) Synthesis and Sequencing Facility. Forward and reverse sequences were imported into Geneious Prime (version 2021.2.2, Biomatters, Ltd, Auckland, New Zealand, https://www.geneious.com), trimmed to remove low-quality reads and primer sequences, and aligned to create a consensus sequence for each specimen. Consensus sequences were compared to the National Center for Biotechnology Information (NCBI) database and reference samples using BLASTn, and final identifications were confirmed if they had > 99% identity to an NCBI sequence. Data were submitted to NCBI’s GenBank and accession numbers were acquired for both ITS2 (OM459737-OM459768) and COI (OM456780-OM456806) sequences.

### Host detection analyses

A host DNA detection assay was created to determine host preference. Primers from Kent & Norris [30], Izadpanah et al. [31], and Kumar et al. [32] were combined into a multiplexed PCR assay to detect individual and mixed blood meals from human, cow, pig, dog, chicken, and goat DNA, producing differential product sizes for each host animal (Additional file 1: Table S1). Each 25 μL PCR reaction consisted of 1 × buffer, 1.0 mM dNTPs, 0.625 units of *Taq* polymerase (New England Biolabs #M0273S), 50 pmol of each primer, and 1.0 μL extracted abdomen DNA (Additional file 1: Table S1). Thermocycler conditions consisted of an initial denaturation of 5 min at 95 °C, followed by 35 cycles at 95 °C for 30 s, 58 °C for 30 s, and 72 °C for 45 s. The final extension step was at 72 °C for 5 min. 12.0 μL of product was then run on an agarose gel for visualization and host determination.

### Detection of sporozoites

Head/thoraces were homogenized at BSPH in a buffer of boiled casein and Nonidet P-40 [18]. Circumsporozoite protein (CSP) ELISAs were performed using controls and protocols from BEI Resources Malaria Research and Reference Reagent Resource Center (MR4) to detect the presence of CSP from *P. falciparum* sporozoites [18, 28]. Samples were run in duplicate pools of five mosquito homogenates for the first ELISA, and then run individually in duplicate if the pool was positive as previously described [3, 29, 33]. Specimens were considered ELISA positive if the absorbance of the well for the individual mosquito was two times the absorbance of a negative insectary control mosquito.

### Risk factor analysis

Ten of the 216 collections were excluded from the analysis because the battery failed on the trap while it was running (n = 6), or the head of household forgot to tie the collection bag, potentially allowing for escaped anophelines (n = 4). A univariate negative binomial generalized linear mixed-effects model was performed with number of *An. gibbinsi* per trap as the outcome for all variables of interest using the glmer.nb function from the MASS package in R. For each model, a household-level random intercept term was included to account for the repeated household visits. Multivariate models were also performed using the glmer.nb function from MASS with a household-level random intercept term. The best model was selected using Akaike information criterion (AIC).

## Results

### *Anopheles gibbinsi* distribution

Each of the enrolled 24 households was visited 9 times, resulting in 216 trap nights. Of the 3091 female anophelines collected in this study, *An. funestus* was collected at the highest abundance (n = 2177, 70.4%), followed by *An. gibbinsi* (n = 453, 14.6%). *Anopheles gibbinsi* were collected from 15 of 24 (62.5%) study households*,* and made up 33.7% (n = 267) animal pen traps, 18.3% (n = 110) traps outdoors where humans gather, and 4.5% (n = 76) indoor sitting room traps (Table 1). Among animal traps, 88% (n = 235) were captured in goat pens (Additional file 1: Table S2), and the majority of *An. gibbinsi* were collected in inland households (n = 447, 98.7%) (Fig. 1).

**Table 1:** Distribution of morphologically identified *An. funestus* and *An. gibbinsi* by location and trap type

Trap location	Species	Trap type			Total
		Indoor n (%)	Animal Pen (%)	Outdoor Gathering n (%)	
Inland	*An. funestus*	1344 (70.6)	203 (10.7)	357 (18.8)	1904
	*An. gibbinsi*	72 (16.1)	265 (59.3)	110 (24.6)	447
	other	59 (13.9)	289 (68.2)	76 (17.9)	424
Lakeside	*An. funestus*	203 (74.4)	27 (9.9)	43 (15.8)	273
	*An. gibbinsi*	4 (66.7)	2 (33.3)	0 (0)	6
	other	14 (37.8)	7 (18.9)	16 (43.2)	37
Total		1696 (54.9)	793 (25.7)	602 (19.5)	3091

### Molecular confirmation

Three samples that had all identifying features (i.e., no missing tarsi, all wing scales present, wings, and proboscis intact) were morphologically confirmed as *An. gibbinsi* at the BSPH and through photographs sent to University of the Witwatersrand (Additional file 1: Figure S1). COI and ITS2 PCRs were performed on these samples and sequenced. The product size (including primers) from the ITS2 PCR for all three samples was 507 base pairs, and the COI product size was 709 base pairs. All three ITS2 sequences were 100% identical to each other, and COI sequences were 98.9% identical to each other. The NCBI BLAST result for all ITS2 and COI sequences was “*An. sp. 6*” with > 99% identity for every specimen (Additional file 1: Table S3). These three samples were used as *An. gibbinsi* reference samples for the remaining study (OM459761-OM459763, OM56804-OM56806).

Of the 17 samples sent to the University of the Witwatersrand, seven were morphologically confirmed as *An. gibbinsi*. Two samples were identified as *Anopheles marshallii* complex, one was identified as *An. funestus*, and six were unable to be confirmed due to loss of legs. ITS2 PCR and sequencing was successful on 5 of 7 that were confirmed as *An. gibbinsi,* and all matched the reference samples with 100% identity.

### Morphological accuracy

Four hundred and fifty-three *An. gibbinsi* were morphologically identified. An ITS2 PCR was performed on all 453 samples, and 443 (97.8%) produced a 507 bp band on electrophoretic analysis, confirming the *An. gibbinsi* identification. Five (1.1%) samples were molecularly identified as *An. funestus s.s.*, two (0.4%) samples had an ITS2 PCR amplicon size of 439 bp and remain unidentified to species level after sequencing, and three (0.7%) samples failed amplification after three attempts. Twenty-four *An. gibbinsi* samples (5.4%) representing all three trap types were selected to undergo ITS2 and COI fragment sequencing to confirm species identity. All ITS2 sequences matched the reference samples with 100% identity, and COI sequences matched reference samples with > 99% identity. All specimens had a NCBI BLAST result of “*An. sp. 6*” with > 99% identity for both COI and ITS2 fragments (Additional file 1: Table S3).

### Host preference and parasite detection

A PCR assay to detect human, goat, cow, dog, pig, and chicken DNA was performed on 417/443 (94.1%) molecularly confirmed *An. gibbinsi* samples to assess host preference. Twenty-six samples were excluded because they did not have an intact abdomen. Host DNA was detected in 83 (19.9%) samples. Sixty three of 417 (15.1%) samples were recorded as visually blood fed during morphology, and host DNA was detected in 54 (85.7%) of those specimens (Additional file 1: Table S4). Additionally, host DNA was detected in 29/354 (8.2%) that were not recorded as visually blooded (Additional file 1: Table S4). Of the *An. gibbinsi* samples positive for host DNA, goat DNA was detected in 71/83 (85.5%) (Table 2). Other hosts detected were pig (n = 7, 8.2%), human (n = 4, 4.8%), and dog (n = 1, 1.2%). The trap type with the highest proportion of specimens with host DNA were from traps placed near animal pens (76/246, 30.8%), followed by traps placed indoors (5/63, 7.9%), and lastly from traps placed near gathering locations (2/103, 1.9%) (Table 2). CSP ELISAs were performed on all 443 samples, but none were positive for sporozoites.

**Table 2:** Host DNA detection by trap type

Trap type	Dog	Goat	Human	Pig	No band	Total
Animal	0	69	0	7	170	246
Outdoor gathering	0	2	0	0	101	103
Indoor	1	0	4	0	63	68
Total	1	71	4	7	334	417

### Risk factor analysis

In the univariate analysis, household level risk factors associated with a higher abundance of *An. gibbinsi* were inland households, animal pens, using an open well or stream/pond for a water source, households with natural wall materials, and not receiving IRS in the previous campaign (October 2018) (Table 3). Increased distance from large streams and increased number of rooms in a household were associated with decreased abundance of *An. gibbinsi* (Table 3). Many of these variables were correlated. For example, inland households were associated with decreased distance to streams, smaller households, and using a stream or pond as a water source, so only one of these variables was included in the final multivariate model. The multivariate model with the lowest AIC included only household location and trap location, and revealed inland households and animal pen traps remained associated with increased counts of *An. gibbinsi* (Table 3)***.***

**Table 3:** Univariate and multivariate results from negative binomial generalized linear mixed-effects model performed with # *An. gibbinsi* per trap as the outcome

Variable	Univariate analysis					Multivariate analysis				
	IRR	SE	95% CI	p-value		IRR	SE	95% CI	p-value	
HH location										
Lakeside										
Inland	84.60	2.20	[20.67, 538.13]	< 0.001	***	86.50	2.25	[20.28, 587.06]	< 0.001	***
Trap type										
Indoor	–	–	-	–		-	-	-	-	
Outdoor gathering	0.98	1.35	[0.52, 1.85]	0.96	ns	1.00	1.38	[0.53, 1.86]	0.989	ns
Animal pen	3.18	1.36	[1.75, 5.83]	< 0.001	***	3.13	1.36	[1.73, 5.72]	< 0.001	***
Distance to stream (km)	0.41	1.41	[0.17, 0.77]	0.01	**	–	–	–	–	
# Rooms in HH	0.43	1.48	[0.18, 0.93]	0.03	*	–	–	–	–	
# People in HH	0.75	1.27	[0.43, 1.19]	0.22	ns	–	–	–	–	
Proportion sleeping under net	0.75	5.27	[0.02, 28.41]	0.86	ns	–	–	–	–	
IRS status										
Yes	–	–	–	–		–	–	–	–	
No	12.7	2.78	[1.73, 136.51]	0.01	**	–	–	–	–	
Water source										
Bore hole	–	–	–	–		–	–	–	–	
Open well	17.5	2.84	[2.26, 135.94]	0.01	**	–	–	–	–	
Steam or pond	46	3	[5.35, 396.04]	< 0.001	***	–	–	–	–	
Wall materials										
Bricks	–	–	–	–		–	–	–	–	
Concrete	2.68	4.81	[0.12, 58.28]	0.53	ns	–	–	–	–	
Natural	28.7	3.69	[2.21, 371.22]	0.01	**	–	–	–	–	
Floor materials										
Finished	–	–	–	–		–	–	–	–	
Natural	1.43	3.27	[0.1, 17.03]	0.761	ns	–	–	–	–	
Roof materials										
Grass	–	–	–	–		–	–	–	–	
Metal	0.99	3.74	[0, 21.95]	0.997	ns	–	–	–	–	
Eaves										
Closed	–	–	–	–		–	–	–	–	
Open	11.6	3.91	[0.8, 168.05]	0.073	ns	–	–	–	–	
Partial	0.42	10.8	[0, 45.2]	0.718	ns	–	–	–	–	
Cooking materials										
Charcoal	–	–	–	–		–	–	–	–	
Coal & charcoal	5.49	4.23	[0.34, 160.66]	0.24	ns	–	–	–	–	
Wood	0.52	3.9	[0.03, 9.04]	0.63	ns	–	–	–	–	
Fire burned last night										
No	–	–	–	–		–	–	–	–	
Yes	0.78	1.38	[0.42, 1.47]	0.43	ns	–	–	–	–	
HH owns cats										
No	–	–	–	–		–	–	–	–	
Yes	3.38	4.38	[0.16, 87.9]	0.41	ns	–	–	–	–	
HH owns chickens										
No	–	–	–	–		–	–	–	–	
Yes	0.88	3.53	[0.06, 12.18]	0.92	ns	–	–	–	–	
HH owns dogs										
No	–	–	–	–		–	–	–	–	
Yes	0.11	3.62	[0.01, 1.3]	0.08	ns	–	–	–	–	
HH owns ducks										
No	–	–	–	–		–	–	–	–	
Yes	0.19	4.51	[0.01, 3.63]	0.26	ns	–	–	–	–	
HH owns goats										
No	–	–	–	–		–	–	–	–	
Yes	1.09	4.56	[0.04, 25.09]	0.96	ns	–	–	–	–	
HH owns pigs										
No	–	–	–	–		–	–	–	–	
Yes	0.7	3.19	[0.06, 9.16]	0.76	ns	–	–	–	–	

*HH *household

* = p value 0.01—0.05; ** = p value 0.001—0.01; *** = p value < 0.001

## Discussion

Prior to this study, *An. gibbinsi* had not been reported in Zambia, very little was known about this species’ foraging behaviours, and no genetic data were associated with this taxon. Perhaps the most unexpected finding is that the ITS2 and COI fragments sequenced from morphologically confirmed *An. gibbinsi* matched with 100% identity to “*An. sp. 6*” data in GenBank from other published work [8, 18]. Identifying *An. sp. 6* as *An. gibbinsi* provides more context for this understudied taxon, as findings from the existing literature can be linked to create a composite understanding of its behaviour and vector potential. For example, *An. sp. 6* was molecularly identified in Nchelenge District in a study from 2016, but morphology did not identify it as *An. gibbinsi*, due to damage to the specimen [18]. In Kenya, specimens morphologically identified as both *An. gambiae* and *An. funestus* were only molecularly identified as *An. sp. 6* [34], illustrating the challenges of identifying these uncommon and often unknown taxa [8]. Generating reference sequences for morphologically confirmed specimens will be invaluable as *Anopheles* species identification continues to incorporate high-throughput molecular techniques and genomic data.

The most common species misidentification was with *An. funestus* (1.1%) followed by an unidentified species (0.4%)*.* This is similar to published reports that found 16/25 (64%) of *An. sp. 6* morphologically identified as *An. funestus,* and 9/25 (36%) identified as *An. gambiae* [34]. Misidentification of anophelines is a common problem when using only morphology, especially when samples are damaged from trapping methods. This underscores the need for more training in morphological identification, and for the development of reliable genetic references for comparison across anopheline taxa.

In this study, 60% of *An. gibbinsi* specimens were caught near animal pens, and goat DNA was detected in 85.5% of all *An. gibbinsi* specimens with detectable host DNA. Additionally, traps near animal pens had the highest proportion of *An. gibbinsi* with host blood compared to trap placements near humans or sheltered/indoor locations, suggesting that this species is largely zoophilic and exophilic. However, 16.3% (n = 73) of specimens were caught indoors, and 5.4% (n = 4) of those were found with a human blood meal. This is similar to findings from the Kenyan highlands where 2/11 (18.%) of *An. sp. 6* harboured human blood meals [34]. While this species appears to be largely zoophilic and exophilic, opportunistic feeding on humans and occasionally indoors may not be unusual, as similarly reported for other secondary and understudied malaria vectors, including *Anopheles coustani, Anopheles rufipes,* and *Anopheles squamosus* [4, 6, 10, 11].

Given the high proportion of goat blood meals in the samples, it is unsurprising that animal pens were associated with a higher abundance of *An. gibbinsi* than traps placed indoors or near where people gather outdoors in both the univariate and multivariate analysis. Inland households, increased proximity to streams, and using an open well/pond were also associated with higher counts of *An. gibbinsi*. These suggest that *An. gibbinsi* may be using slow moving streams and rivers as breeding sites in Nchelenge District during the dry season. However, considering that inland households were specifically selected because of their proximity to potential breeding sites, it is possible there is another explanation for these associations that was not captured in this study. Additionally, walls made from natural materials were also associated with higher *An. gibbinsi* counts. Natural wall materials and using a stream or pond as a water source compared to a bore hole may indicative of lower socioeconomic status or temporary housing, which may also impact mosquito densities.

This study did not detect any *An. gibbinsi* specimens positive for *P. falciparum* sporozoites, but parasite-positive *An. sp. 6* (2/27, 7.4%) were reported from the Kenyan highlands found using a multiplexed qPCR method [34]. Additionally, another study from the Kenyan highlands found 7.7% of morphologically-identified *An. gibbinsi* positive for sporozoites by CSP ELISA [2]*.* Given the high transmission in Nchelenge District and potential for *An. gibbinsi* to serve as a vector, it is important that this species be included in ongoing and future malaria surveillance. Future studies should also assess vector competence with live field-captured mosquitoes to more fully understand the capacity for this species to transmit malaria parasites.

## Conclusion

This study documented *An. gibbinsi* as an anopheline species present in the dry season of 2019 in Nchelenge District, Zambia: the first report of this anopheline species from Zambia. Comparison of COI and ITS2 sequences to NCBI’s GenBank database revealed > 99% identity to *An. sp. 6,* which has been implicated in malaria transmission in Kenya [2, 34]. Most specimens were captured near animal pens, and host DNA detection revealed a propensity for goats. Although this finding may be skewed by the collection method, composite data suggest that this species is largely zoophilic and exophilic. However, 5% of specimens with detected host DNA had fed on humans, indicating that this potential vector species is likely to ingest human malaria parasites. The vector competence of *An. gibbinsi*, as supported by reports from Kenya, suggest that this species should continue to be monitored in Nchelenge District. Importantly, this study also provides genetic references for *An. gibbinsi,* which will help inform future studies as molecular identification and verification become more common in malaria entomology.

## Supplementary Information

**Additional file 1: Figure S1. **Identifying features (Coetzee 2020) of an *An. gibbinsi *sample that was molecularly confirmed as *An. species 6*. **Table S1**. Primers included in Host DNA PCR. **Table S2**. Sequenced *An. gibbinsi *samples. **Table S3.** Host DNA detection by visually blooded status.

## Data Availability

The datasets used in the present study are not publicly available to protect the confidentiality and privacy of study participants, but are available from the corresponding author upon appropriate reasonable request and approval from the corresponding national research and ethics committee.

## Abbreviations

IRS: Indoor residual spray
ITN: Insecticide treated net
CSP: Circumsporozoite protein
CDC LT: Center for disease control light trap
DRC: Democratic Republic of Congo
BSPH: Johns Hopkins Bloomberg School of Public Health
ITS2: Internal transcribed spacer 2
COI: Cytochrome oxidase I
PCR: Polymerase chain reaction
JHMI: Johns Hopkins Medical Institute
MR4: Malaria Research and Reference Reagent Resource Center
ELISA: Enzyme-linked immunoabsorbant assay
AIC: Akaike information criterion
JHMRI: Johns Hopkins Malaria Research Institute
NCBI: National Center for Biotechnology Information
