# Supplementary material for: Expanded geographic distribution and host preference of Anopheles gibbinsi (Anopheles species 6) in northern Zambia
Source: Malar J. 2022 Jul 3;21:211. doi: 10.1186/s12936-022-04231-5 (PMC9250713; doi:10.1186/s12936-022-04231-5)
Supplement: Supplementary file 1 — Additional file 1: Figure S1. Identifying features (Coetzee 2020) of an An. gibbinsi sample that was molecularly confirmed as An. species 6. Table S1. Primers included in Host DNA PCR. Table S2. Sequenced An. gibbinsi samples. Table S3. Host DNA detection by visually blooded status. [file 12936_2022_4231_MOESM1_ESM.pptx]

## Slide 1
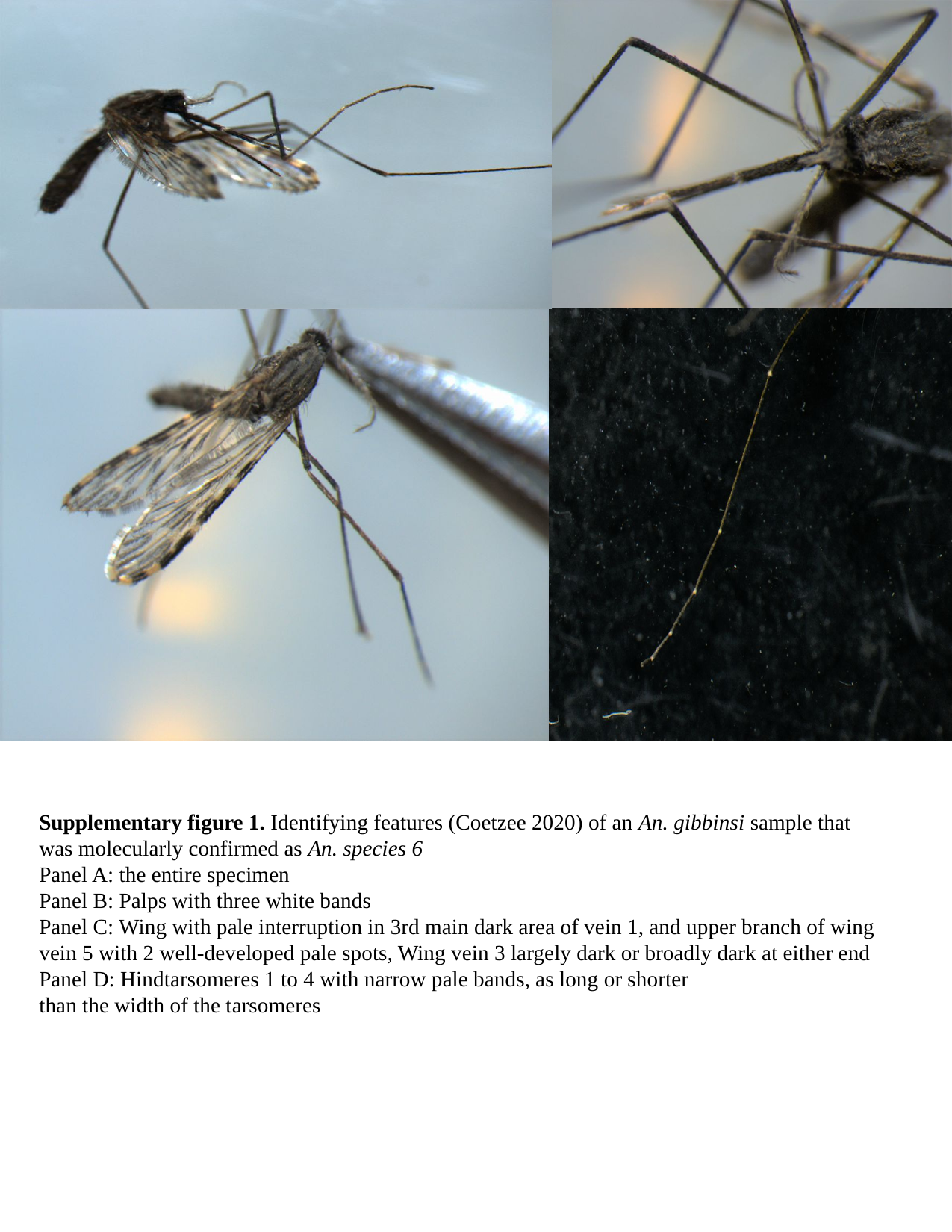

Supplementary figure 1. Identifying features (Coetzee 2020) of an An. gibbinsi sample that was molecularly confirmed as An. species 6
Panel A: the entire specimen
Panel B: Palps with three white bands
Panel C: Wing with pale interruption in 3rd main dark area of vein 1, and upper branch of wing vein 5 with 2 well-developed pale spots, Wing vein 3 largely dark or broadly dark at either end Panel D: Hindtarsomeres 1 to 4 with narrow pale bands, as long or shorter
than the width of the tarsomeres

## Slide 2
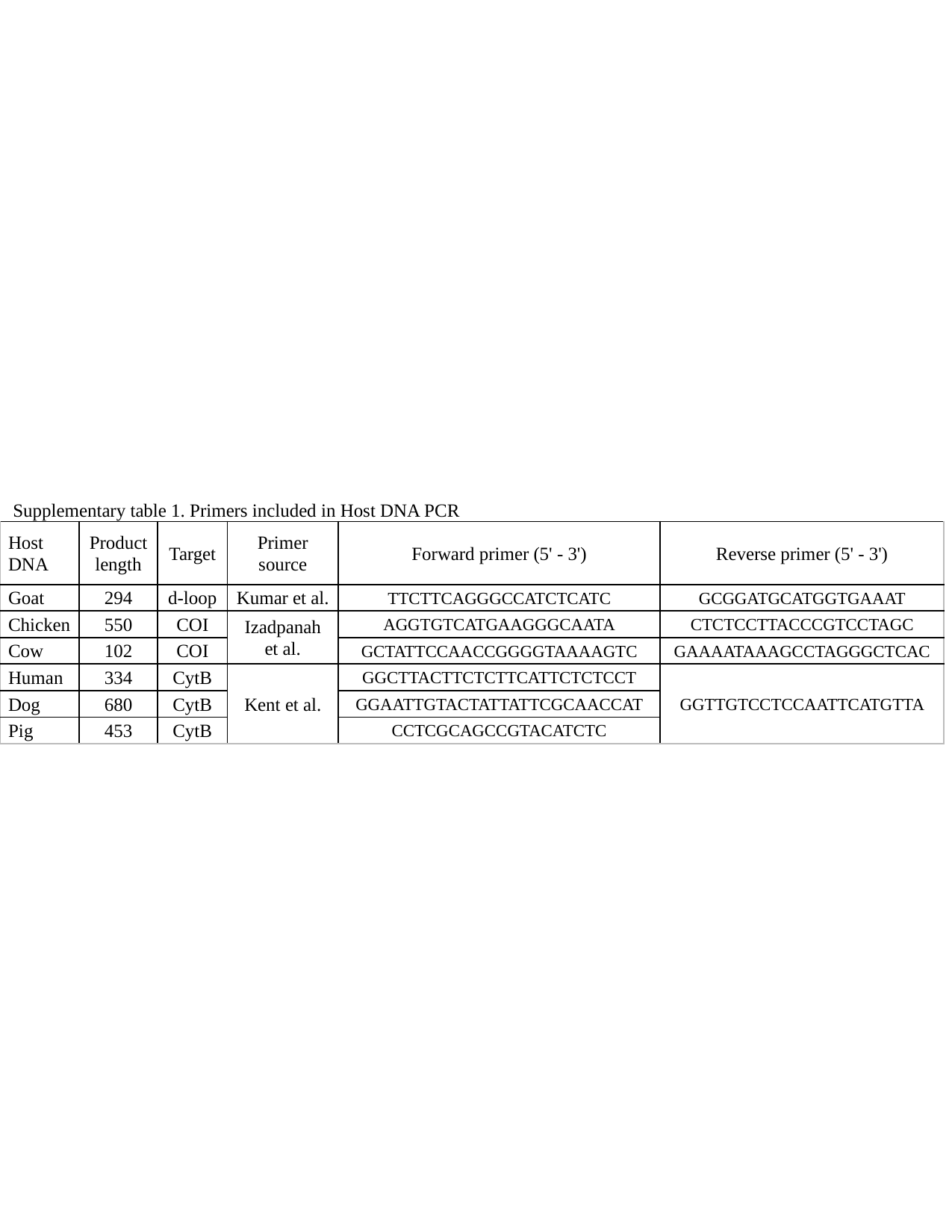

| Supplementary table 1. Primers included in Host DNA PCR | | | | | |
| --- | --- | --- | --- | --- | --- |
| Host DNA | Product length | Target | Primer source | Forward primer (5' - 3') | Reverse primer (5' - 3') |
| Goat | 294 | d-loop | Kumar et al. | TTCTTCAGGGCCATCTCATC | GCGGATGCATGGTGAAAT |
| Chicken | 550 | COI | Izadpanah et al. | AGGTGTCATGAAGGGCAATA | CTCTCCTTACCCGTCCTAGC |
| Cow | 102 | COI | | GCTATTCCAACCGGGGTAAAAGTC | GAAAATAAAGCCTAGGGCTCAC |
| Human | 334 | CytB | Kent et al. | GGCTTACTTCTCTTCATTCTCTCCT | GGTTGTCCTCCAATTCATGTTA |
| Dog | 680 | CytB | | GGAATTGTACTATTATTCGCAACCAT | |
| Pig | 453 | CytB | | CCTCGCAGCCGTACATCTC | |

## Slide 3
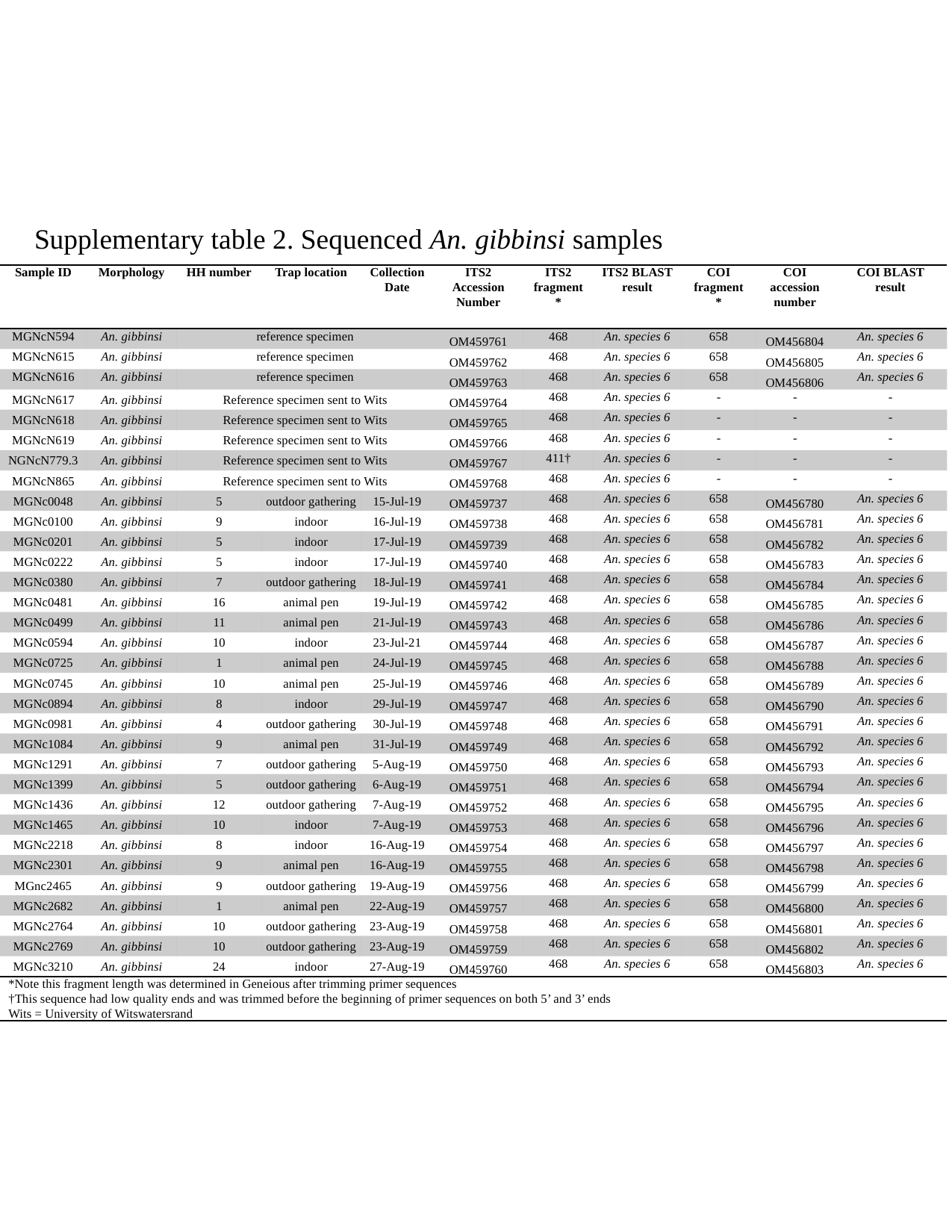

Supplementary table 2. Sequenced An. gibbinsi samples
| Sample ID | Morphology | HH number | Trap location | Collection Date | ITS2 Accession Number | ITS2 fragment\* | ITS2 BLAST result | COI fragment \* | COI accession number | COI BLAST result |
| --- | --- | --- | --- | --- | --- | --- | --- | --- | --- | --- |
| MGNcN594 | An. gibbinsi | reference specimen | | | OM459761 | 468 | An. species 6 | 658 | OM456804 | An. species 6 |
| MGNcN615 | An. gibbinsi | reference specimen | | | OM459762 | 468 | An. species 6 | 658 | OM456805 | An. species 6 |
| MGNcN616 | An. gibbinsi | reference specimen | | | OM459763 | 468 | An. species 6 | 658 | OM456806 | An. species 6 |
| MGNcN617 | An. gibbinsi | Reference specimen sent to Wits | | | OM459764 | 468 | An. species 6 | - | - | - |
| MGNcN618 | An. gibbinsi | Reference specimen sent to Wits | | | OM459765 | 468 | An. species 6 | - | - | - |
| MGNcN619 | An. gibbinsi | Reference specimen sent to Wits | | | OM459766 | 468 | An. species 6 | - | - | - |
| NGNcN779.3 | An. gibbinsi | Reference specimen sent to Wits | | | OM459767 | 411† | An. species 6 | - | - | - |
| MGNcN865 | An. gibbinsi | Reference specimen sent to Wits | | | OM459768 | 468 | An. species 6 | - | - | - |
| MGNc0048 | An. gibbinsi | 5 | outdoor gathering | 15-Jul-19 | OM459737 | 468 | An. species 6 | 658 | OM456780 | An. species 6 |
| MGNc0100 | An. gibbinsi | 9 | indoor | 16-Jul-19 | OM459738 | 468 | An. species 6 | 658 | OM456781 | An. species 6 |
| MGNc0201 | An. gibbinsi | 5 | indoor | 17-Jul-19 | OM459739 | 468 | An. species 6 | 658 | OM456782 | An. species 6 |
| MGNc0222 | An. gibbinsi | 5 | indoor | 17-Jul-19 | OM459740 | 468 | An. species 6 | 658 | OM456783 | An. species 6 |
| MGNc0380 | An. gibbinsi | 7 | outdoor gathering | 18-Jul-19 | OM459741 | 468 | An. species 6 | 658 | OM456784 | An. species 6 |
| MGNc0481 | An. gibbinsi | 16 | animal pen | 19-Jul-19 | OM459742 | 468 | An. species 6 | 658 | OM456785 | An. species 6 |
| MGNc0499 | An. gibbinsi | 11 | animal pen | 21-Jul-19 | OM459743 | 468 | An. species 6 | 658 | OM456786 | An. species 6 |
| MGNc0594 | An. gibbinsi | 10 | indoor | 23-Jul-21 | OM459744 | 468 | An. species 6 | 658 | OM456787 | An. species 6 |
| MGNc0725 | An. gibbinsi | 1 | animal pen | 24-Jul-19 | OM459745 | 468 | An. species 6 | 658 | OM456788 | An. species 6 |
| MGNc0745 | An. gibbinsi | 10 | animal pen | 25-Jul-19 | OM459746 | 468 | An. species 6 | 658 | OM456789 | An. species 6 |
| MGNc0894 | An. gibbinsi | 8 | indoor | 29-Jul-19 | OM459747 | 468 | An. species 6 | 658 | OM456790 | An. species 6 |
| MGNc0981 | An. gibbinsi | 4 | outdoor gathering | 30-Jul-19 | OM459748 | 468 | An. species 6 | 658 | OM456791 | An. species 6 |
| MGNc1084 | An. gibbinsi | 9 | animal pen | 31-Jul-19 | OM459749 | 468 | An. species 6 | 658 | OM456792 | An. species 6 |
| MGNc1291 | An. gibbinsi | 7 | outdoor gathering | 5-Aug-19 | OM459750 | 468 | An. species 6 | 658 | OM456793 | An. species 6 |
| MGNc1399 | An. gibbinsi | 5 | outdoor gathering | 6-Aug-19 | OM459751 | 468 | An. species 6 | 658 | OM456794 | An. species 6 |
| MGNc1436 | An. gibbinsi | 12 | outdoor gathering | 7-Aug-19 | OM459752 | 468 | An. species 6 | 658 | OM456795 | An. species 6 |
| MGNc1465 | An. gibbinsi | 10 | indoor | 7-Aug-19 | OM459753 | 468 | An. species 6 | 658 | OM456796 | An. species 6 |
| MGNc2218 | An. gibbinsi | 8 | indoor | 16-Aug-19 | OM459754 | 468 | An. species 6 | 658 | OM456797 | An. species 6 |
| MGNc2301 | An. gibbinsi | 9 | animal pen | 16-Aug-19 | OM459755 | 468 | An. species 6 | 658 | OM456798 | An. species 6 |
| MGnc2465 | An. gibbinsi | 9 | outdoor gathering | 19-Aug-19 | OM459756 | 468 | An. species 6 | 658 | OM456799 | An. species 6 |
| MGNc2682 | An. gibbinsi | 1 | animal pen | 22-Aug-19 | OM459757 | 468 | An. species 6 | 658 | OM456800 | An. species 6 |
| MGNc2764 | An. gibbinsi | 10 | outdoor gathering | 23-Aug-19 | OM459758 | 468 | An. species 6 | 658 | OM456801 | An. species 6 |
| MGNc2769 | An. gibbinsi | 10 | outdoor gathering | 23-Aug-19 | OM459759 | 468 | An. species 6 | 658 | OM456802 | An. species 6 |
| MGNc3210 | An. gibbinsi | 24 | indoor | 27-Aug-19 | OM459760 | 468 | An. species 6 | 658 | OM456803 | An. species 6 |
| \*Note this fragment length was determined in Geneious after trimming primer sequences †This sequence had low quality ends and was trimmed before the beginning of primer sequences on both 5’ and 3’ ends Wits = University of Witswatersrand | | | | | | | | | | |

## Slide 4
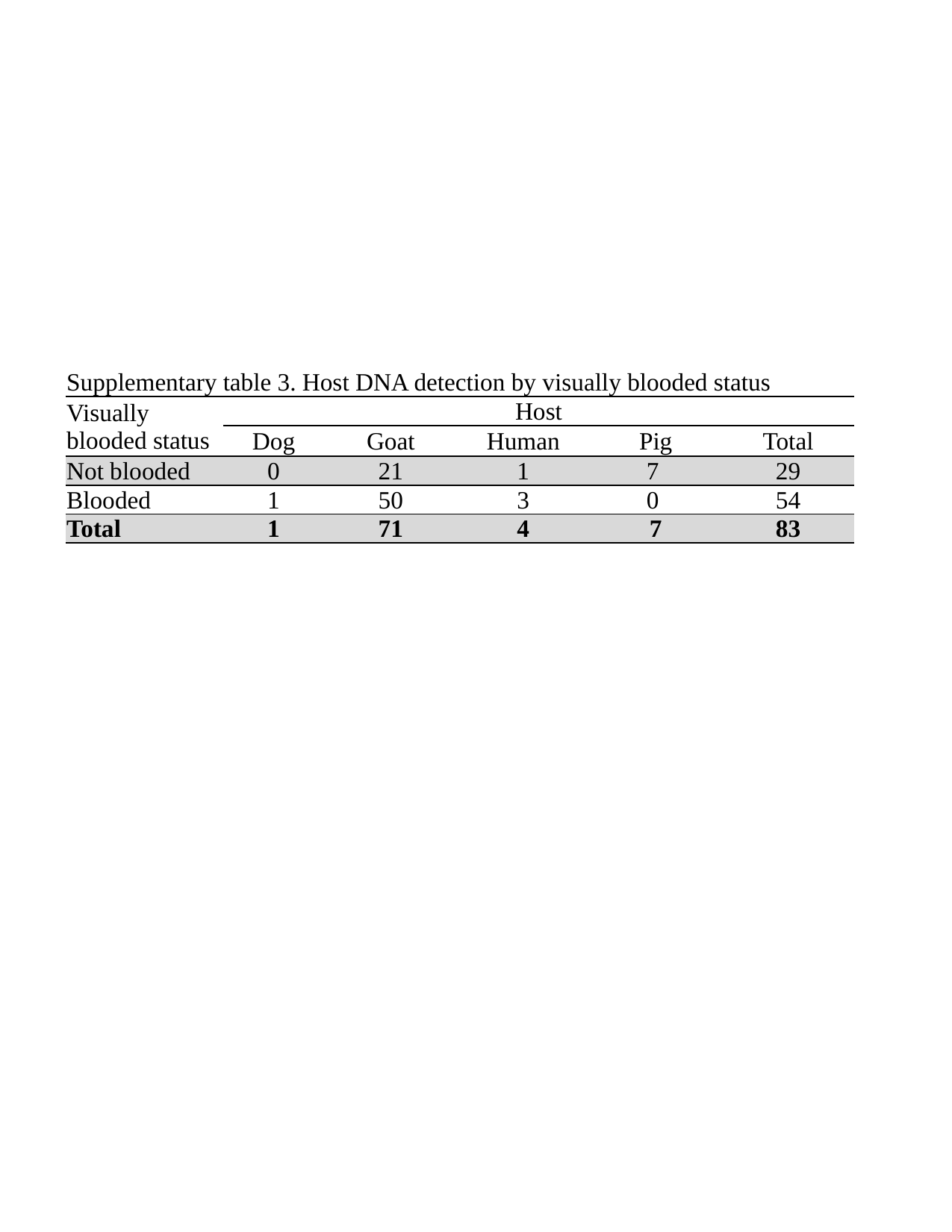

| Supplementary table 3. Host DNA detection by visually blooded status | | | | | |
| --- | --- | --- | --- | --- | --- |
| Visually blooded status | Host | | | | |
| | Dog | Goat | Human | Pig | Total |
| Not blooded | 0 | 21 | 1 | 7 | 29 |
| Blooded | 1 | 50 | 3 | 0 | 54 |
| Total | 1 | 71 | 4 | 7 | 83 |
